# Characterization of the partial volume effect along the axial field-of-view of the Biograph Vision Quadra total-body PET/CT system for multiple isotopes

**DOI:** 10.1186/s40658-023-00554-7

**Published:** 2023-05-27

**Authors:** Julia G. Mannheim, Ivo Rausch, Maurizio Conti, Christian la Fougère, Fabian P. Schmidt

**Affiliations:** 1 Department of Preclinical Imaging and Radiopharmacy, Werner Siemens Imaging Center, Eberhard-Karls University Tuebingen, Roentgenweg 13, 72076 Tuebingen, Germany; 2 Cluster of Excellence iFIT (EXC 2180) “Image Guided and Functionally Instructed Tumor Therapies”, University of Tuebingen, Tuebingen, Germany; 3 QIMP Team, Center for Medical Physics and Biomedical Engineering, Medical University of Vienna, Vienna, Austria; 4 Molecular Imaging, Siemens Medical Solutions USA, Inc., Knoxville, TN USA; 5 Department of Nuclear Medicine and Clinical Molecular Imaging, University Hospital Tuebingen, Tuebingen, Germany

**Keywords:** Total-body PET, Partial volume effect, Voxel noise, Biograph Vision Quadra, Extended axial FOV

## Abstract

**Background:**

Total-body PET scanners with axial field of views (FOVs) longer than 1 m enable new applications to study multiple organs (e.g., the brain-gut-axis) simultaneously. As the spatial resolution and the associated partial volume effect (PVE) can vary significantly along the FOV, detailed knowledge of the contrast recovery coefficients (CRCs) is a prerequisite for image analysis and interpretation of quantitative results. The aim of this study was to determine the CRCs, as well as voxel noise, for multiple isotopes throughout the 1.06 m axial FOV of the Biograph Vision Quadra PET/CT system (Siemens Healthineers).

**Materials and Methods:**

Cylindrical phantoms equipped with three different sphere sizes (inner diameters 7.86 mm, 28 and 37 mm) were utilized for the PVE evaluation. The 7.86 mm sphere was filled with F-18 (8:1 and 4:1), Ga-68 (8:1) and Zr-89 (8:1). The 28 mm and 37 mm spheres were filled with F-18 (8:1). Background concentration in the respective phantoms was of ~ 3 kBq/ml. The phantoms were measured at multiple positions in the FOV (axial: 0, 10, 20, 30, 40 and 50 cm, transaxial: 0, 10, 20 cm). The data were reconstructed with the standard clinical protocol, including PSF correction and TOF information with up to 10 iterations for maximum ring differences (MRDs) of 85 and 322; CRCs, as well as voxel noise levels, were determined for each position.

**Results:**

F-18 CRCs (SBR 8:1 and 4:1) of the 7.86 mm sphere decreased up to 18% from the center FOV (cFOV) toward the transaxial edge and increased up to 17% toward the axial edge. Noise levels were below 15% for the default clinical reconstruction parameters. The larger spheres exhibited a similar pattern. Zr-89 revealed ~ 10% lower CRCs than F-18 but larger noise (9.1% (F-18), 19.1% (Zr-89); iteration 4, cFOV) for the default reconstruction. Zr-89 noise levels in the cFOV significantly decreased (~ 28%) when reconstructing the data with MRD322 compared with MRD85 along with a slight decrease in CRC values. Ga-68 exhibited the lowest CRCs for the three isotopes and noise characteristics comparable to those of F-18.

**Conclusions:**

Distinct differences in the PVE within the FOV were detected for clinically relevant isotopes F-18, Ga-68 and Zr-89, as well as for different sphere sizes. Depending on the positions inside the FOV, the sphere-to-background ratios, count statistics and isotope used, this can result in an up to 50% difference between CRCs. Hence, these changes in PVE can significantly affect the quantitative analysis of patient data. MRD322 resulted in slightly lower CRC values, especially in the center FOV, whereas the voxel noise significantly decreased compared with MRD85.

**Supplementary Information:**

The online version contains supplementary material available at 10.1186/s40658-023-00554-7.

## Background

Combined positron emission tomography (PET)/computed tomography (CT) systems have evolved to a standard of care imaging diagnosis tool over the last decades with applications, e.g., in oncology, cardiology and neurology [1, 2]. However, PET imaging in particular tends to be constrained by the available axial field of view (FOV) with typical lengths of 16–30 cm limiting the sensitivity to approximately 0.6–2% [1, 3]. This limitation requires either higher injection doses to ensure adequate count statistics while increasing patient radiation doses or an increase in scan time resulting in both patient discomfort and limited patient throughput [4]. The short axial FOVs also require PET acquisitions at multiple bed positions to perform whole-body imaging. This can be achieved either by stitching images of respective bed positions or by continuously moving the bed [5]. However, neither of those approaches permits simultaneous observation of tracer kinetics in multiple organs [3].

These limitations can be overcome with the recent introduction of total-body (TB) PET scanners with axial FOVs of 70 cm or more [6, 7], which according to Surti et al*.* is sufficient to capture all major organs of the body within one bed position [1]. Vandenberghe et al. [3] estimated a 10–40 × gain in sensitivity for multiorgan imaging in oncological applications in TB PET scanners compared with conventional PET/CT scanners.

This increase in sensitivity can be exploited to either reduce the injected activity or to decrease the scan time without sacrificing on the image quality [1]. This improvement fosters new applications, e.g., imaging of novel tracers such as Zr-89-labeled antibodies with slow kinetics, dual-tracer imaging of two distinct biological targets or low-count imaging of Y-90 [1, 8]. Furthermore, the significant increase in the signal-to-noise ratio (SNR) allows short time frames to capture fast kinetic tracers. The combination with simultaneous dynamic imaging of multiple organs enables new application areas as well as advanced kinetic modeling.

However, the spatial resolution and the associated partial volume effect (PVE) can vary significantly along the extended axial FOV of TB PET systems, especially if the parallax error is not accounted for by depth-of-interaction correction [9]. The PVE is inherent to PET imaging and can significantly affect the quantification accuracy by underestimating the true activity within voxels due to spill-over in adjacent regions [10, 11]. Therefore, detailed knowledge of contrast recovery coefficients (CRCs) and hence the PVE is essential.

Over recent decades, there has been emerging evidence that many diseases involve the interplay of multiple organs, e.g., the brain-to-gut axis, liver-to-heart axis and heart-to-brain axis [4, 12, 13]. When exploring such axis interactions within the extended axial FOV of TB PET scanners, organs of interest might be differently affected by the PVE due to their size, shape and target-to-background ratios but also based on their positions within the FOV. Furthermore, the isotope-specific positron range also affects the activity recovery in the FOV. Currently, there is no clinically established PVE correction method for PET data available [10, 14]; therefore, the variation in the quantitative uptake due to the previously mentioned parameters may impact scientific and clinical interpretation of the measurements [11]. Hence, it is desirable to investigate the PVE in detail for TB PET scanners.

The aim of this study is to fully characterize the extended axial FOV of the Biograph Vision Quadra PET/CT (Siemens Healthineers, Knoxville, TN, USA), a commercially available clinical scanner with an axial FOV of 1.06 m, with respect to the PVE and voxel noise. CRCs and coefficients of variation (CVs) as a measure of voxel noise were determined for different sphere sizes, contrast ratios, multiple isotopes and at multiple positions within the FOV. Hence, our evaluation goes far beyond the National Electrical Manufacturers Association (NEMA) NU 2–2018 performance evaluation protocol [15] and the European Association of Nuclear Medicine (EANM) Research Ltd. (EARL) requirements [16, 17], which require the phantom to be positioned only in the center FOV (cFOV) filled with F-18 and a contrast ratio of 4:1. However, detailed knowledge of the PVE and voxel noise change along the entire FOV for multiple clinically relevant isotopes will support and enhance the interpretation of quantitative results for TB PET scanners.

Our evaluation is performed based on the vendors’ current clinically available maximum ring difference (MRD) of 85 (acceptance angle 18°). Compared with the maximum possible ring difference of 322 (acceptance angle 52°), MRD85 does not utilize all possible lines of responses within the extended axial FOV resulting in a ~ 2.5-fold lower peak sensitivity in the cFOV (MRD85: 200 cps/MBq, MRD322: 549 cps/MBq) [7]. To assess the impact of MRD322, we reconstructed selected datasets using an investigational research prototype version and evaluated the impact on the CRCs and CVs, as the increase in sensitivity might affect these measures. Therefore, our evaluation focuses on the current available reconstruction for clinical use with MRD85 and provides an outlook on the usage of MRD322.

This study differs from the recently published study by Rausch et al. [18] as it further investigates the CRCs and voxel noise at multiple positions within the FOV with both axial and transaxial offsets for multiple isotopes (F-18, Zr-89 and Ga-68), different acceptance angles and number of iterations for image reconstruction, whereas Rausch et al. investigated the CRCs at four axial positions focusing on F-18 for the standard clinical reconstruction protocol using an Ordinary Poisson ordered subsets expectation maximization (OP-OSEM) algorithm with four iterations and an acceptance angle of 18° [18]. Hence, our study provides additional data to fully characterize the entire FOV of the Biograph Vision Quadra in regards to CRCs and voxel noise.

## Methods

### System description

The Biograph Vision Quadra PET/CT scanner (Siemens Healthineers) is based on block detectors arranged in a ring design with 3.2 × 3.2 × 20 mm^3^ lutetium oxyorthosilicate (LSO) crystals coupled to silicon photomultipliers (SiPMs) spanning an axial FOV of 1.06 m and a transaxial FOV of 78 cm [7]. The scanner’s time-of-flight resolution is 228 ps, the radial spatial resolution with MRD85 is 3.35 mm (at 1/2 of the axial FOV (53.0 cm) and radial offset of 1 cm), and a total sensitivity of 83 cps/kBq (MRD85) and 175 cps/kBq (MRD322) is reported in the cFOV [7].

### Phantom measurements

Two different sets of phantoms were used to determine the PVE along the FOV. First, a cylindrical preclinical micro hollow sphere phantom (volume ~ 127 mL) equipped with a single fillable sphere (inner diameter: 7.86 mm, volume: 250 µL) was used. Second, a 3D-printed cylindrical phantom (volume ~ 675 mL) was equipped with individual spheres (inner diameters: 28 and 37 mm, volume: 11.5 and 26.5 mL, respectively, from the image quality phantom of the NEMA NU-2 2018 standard [15]).

The spheres and background were filled with F-18, Ga-68 and Zr-89. Details on the activity concentration and the sphere-to-background ratios (SBRs) are listed in Table 1. SBRs were calculated according the following equation:

1 $${\text{sphere}} - {\text{to}} - {\text{background}}\;{\text{ ratios}} \,\left( {{\text{SBRs}}} \right) = \frac{{\left( {{\text{activity}}\;{\text{concentration}}_{{{\text{sphere}}}} \left[ {{\text{MBq}}/{\text{ml}}} \right]} \right)}}{{({\text{activity}}\;{\text{concentration}}_{{{\text{background}}}} \left[ {{\text{MBq}}/{\text{ml}}} \right])}}$$

**Table 1:** Sphere-to-background ratios (SBRs) and activity concentrations (ACs) in the background for the respective phantom measurements

Sphere diameter [mm]	^18^F		^68^ Ga		^89^Zr	
	SBR	AC BG [kBq/ml]	SBR	AC BG [kBq/ml]	SBR	AC BG [kBq/ml]
7.86	7.7: 1	3.0	8.3: 1	3.0	7.5: 1	2.6
	4.0: 1	3.0				
28	8.1: 1	3.0	n. a.			
37	8.1:1	3.0	n. a.			

**Fig. 1:**
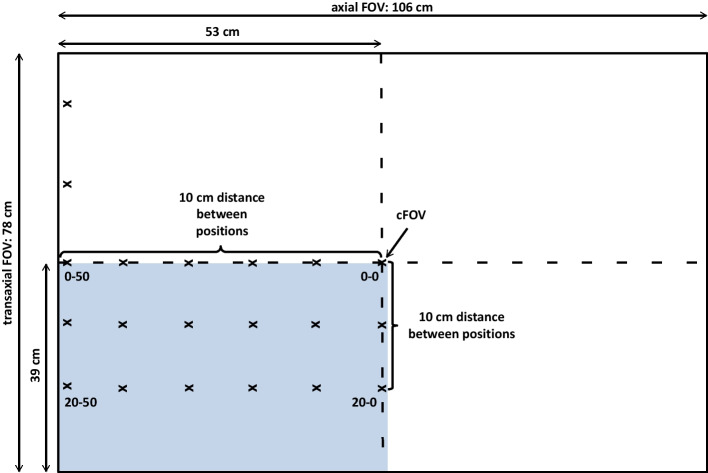
Phantom positions along the axial and transaxial FOV. Note that only a quarter of the FOV was scanned, and asymmetry was assumed for the remaining FOV

The phantoms were mounted on a fixture attached to the patient bed enabling precise movement in the transaxial direction, whereas axial movement was performed by moving the patient bed accordingly. The phantoms were measured at multiple positions along the transaxial and axial FOV (Fig. 1) to determine CRC maps along the entire FOV.

Listmode data were acquired for 3 min for the cFOV position and prolonged based on the decay of the respective isotopes for the subsequent positions. All acquisitions were performed by recording all possible lines of responses (LORs) with MRD322. The current clinical default reconstruction is using MRD85 and the standard image reconstruction recommended by the vendor was utilized (OP-OSEM algorithm with 4 iterations and 5 subsets, point-spread-function (PSF) modeling, TOF information, matrix: 440 × 440 × 645 resulting in a reconstructed voxel size of 1.65 × 1.65 × 1.65 mm^3^). A standard-of-care diagnostic CT scan was performed prior to the emission scan for each phantom position to correct for attenuation (120 kVp tube potential, automatic tube current modulation with 210 mAs ref.).

Furthermore, the acquired phantom data at the scanner's center FOV (position 0–0), transaxial offset of 20 cm (position 20–0), axial offset of 50 cm (position 0–50) and transaxial offset of 20 cm along with an axial offset of 50 cm (position 20–50) were reconstructed with up to 10 iterations to investigate the voxel noise characteristics as well as the contrast recovery as a function of iterations for the different sphere sizes and isotopes at these selected positions within the FOV.

To assess the impact of the MRD on the CRCs, the F-18 data of the 7.86 mm and 37 mm spheres (all positions) were reconstructed with MRD322 using a dedicated workstation and an investigational research prototype version (e7 tools, VR20, Siemens Healthineers). Furthermore, voxel noise was determined for the cFOV position for different frame durations (30 s, 60 s, 120 s and 180 s) to investigate the impact of the count statistics on the noise for both MRDs. The influence of the increased sensitivity on the voxel noise by using MRD322 for reconstruction was also determined for the Zr-89 7.86 mm sphere data for the four selected positions within the FOV (see above) for iterations 1 to 10.

### Phantom data analysis

Spherical volumes of interest (VOIs) based on the actual volume of the respective spheres were placed on the CT images using the software tool pmod (version 4.2, PMOD Technologies LLC, Zurich, Switzerland) and transferred to the coregistered PET images. Background VOIs were placed in the homogeneous background region of the phantoms. The CRC for each position along the FOV was calculated according to the following equation:

2 $${\text{contrast}}\;{\text{ recovery}} \;{\text{coefficient}}\, \left( {{\text{CRC}}} \right) = \frac{{\left( {{\text{contrast}} \;{\text{ratio}}_{{{\text{measured}}}} - 1} \right)}}{{({\text{contrast}}\; {\text{ratio}}_{{{\text{true}}}} - 1)}}$$

Voxel noise in % was calculated by determining the CV for voxel values within a uniform background region in the phantom according to the following equation:

3 $${\text{coefficient}}\;{\text{of}}\;{\text{variation}}\, \left( {{\text{CV}},\% } \right) = \frac{{{\text{standard}}\;{\text{deviation}}_{{{\text{background}}}} }}{{{\text{concentration}}_{{{\text{background}}}} }} \times 100\%$$

## Results

### Contrast recovery coefficient maps

**Fig. 2:**
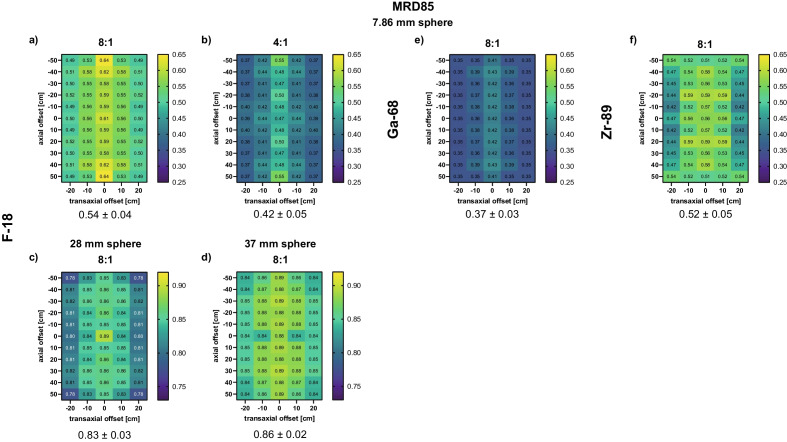
CRC maps along the FOV for F-18 (7.86 mm sphere: SBR 8:1 (**a**), SBR 4:1 (**b**); 28 mm sphere: SBR 8:1 (**c**); 37 mm sphere: SBR 8:1 (**d**)), Ga-68 (7.86 mm sphere: SBR 8:1 (**e**)) and Zr-89 (7.86 mm sphere: SBR 8:1 (**f**)). Note that for the 7.86 mm sphere maps, the same scale was used, whereas a different scale was used for the 37 and 28 mm spheres. Data were reconstructed with MRD85. The mean ± standard deviation was determined over all positions for the respective isotope

Figure 2a and b depicts the 2D profiles of the CRCs along the transaxial and axial FOV for SBRs of 8:1 and 4:1 using the 7.86 mm sphere filled with F-18 and reconstructed with MRD85, respectively. As expected, higher CRCs were detected for the 8:1 SBR (mean ± standard deviation over all positions: 0.54 ± 0.04) compared with a 4:1 SBR (0.42 ± 0.05). However, a similar trend along the FOV was detected for both SBRs. In particular, the CRCs decreased with increasing transaxial offset compared to the cFOV (SBR 8:1: 0.61 to 0.50; SBR 4:1: 0.47 to 0.39). A trend toward increased CRCs when moving toward the axial edge of the cFOV (SBR 8:1: 0.61 to 0.64; SBR 4:1: 0.47 to 0.55) was detected. At transaxial offsets of 10 and 20 cm, respectively, the CRCs remained relatively stable along the axial FOV.

The CRCs increased with larger sphere diameters (28 mm: 0.83 ± 0.03; 37 mm: 0.86 ± 0.02; Fig. 2c and d). Along the transaxial FOV, a similar pattern to that observed for the 7.86 mm sphere filled with F-18 was determined, with CRCs decreasing from the cFOV towards the transaxial FOV edge (28 mm: 0.89 to 0.80; 37 mm: 0.88 to 0.84).

Lower CRCs were detected for the 7.86 mm sphere filled with Ga-68 (0.37 ± 0.03), whereas Zr-89 (0.52 ± 0.05) exhibited similar CRCs compared to F-18 SBR 8:1.

### Voxel noise at four positions within the FOV

**Fig. 3:**
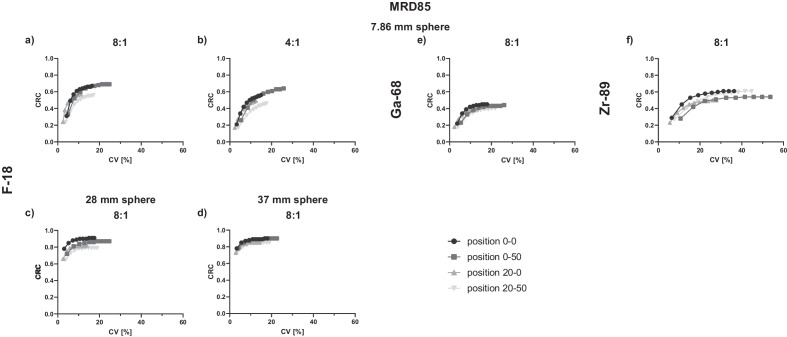
CRCs as a function of CV [%] for F-18 (7.86 mm sphere: SBR 8:1 (**a**), SBR 4:1 (**b**); 28 mm sphere: SBR 8:1 (**c**); 37 mm sphere: SBR 8:1 (**d**)), Ga-68 (7.86 mm sphere: SBR 8:1 (**e**)) and Zr-89 (7.86 mm sphere: SBR 8:1 (**f**)) for 4 different positions within the FOV. Position 0–0: axial and transaxial cFOV; position 0–50: axial offset of 50 cm, transaxial cFOV; position 20–0: axial cFOV, transaxial offset of 20 cm; position 20–50: axial offset of 50 cm, transaxial offset of 20 cm. Each point represents one iteration, and the number of iterations increases from left to right. Data were reconstructed with MRD85

Figure 3a (SBR 8:1) and b (SBR 4:1) depicts the CRCs as a function of CV for the smallest sphere with F-18 and MRD85 reconstruction at positions 0–0, 20–0, 0–50 and 20–50. The highest CVs (24.8% and 25.8%) along with the highest CRCs (0.69 and 0.64) were determined for 10 iterations and at position 0–50 for SBRs of 8:1 and 4:1, respectively. The lowest CVs for each iteration were detected for the position 20–0 along with lower CRCs. The CRCs remained relatively stable from 4 iterations on for all investigated positions, whereas the noise continuously increased with more iterations, e.g., from 9.1% and 13.3% (4 iterations) to 16.3% and 24.8% (10 iterations) for positions 0–0 and 0–50 with SBR 8:1, respectively.

The larger spheres filled with F-18 (Fig. 3c and d) exhibited comparable noise characteristics.

For Ga-68, noise characteristics similar to those for the 7.86 mm sphere filled with F-18 were obtained (Fig. 3e). Zr-89 revealed the largest overall noise (Fig. 3f) with a maximum CV of 53.7% compared with F-18 (8:1: 24.8%, 4:1: 25.8%) and Ga-68 (26.1%).

### Comparison MRD85 to MRD322: recovery maps and voxel noise

**Fig. 4:**
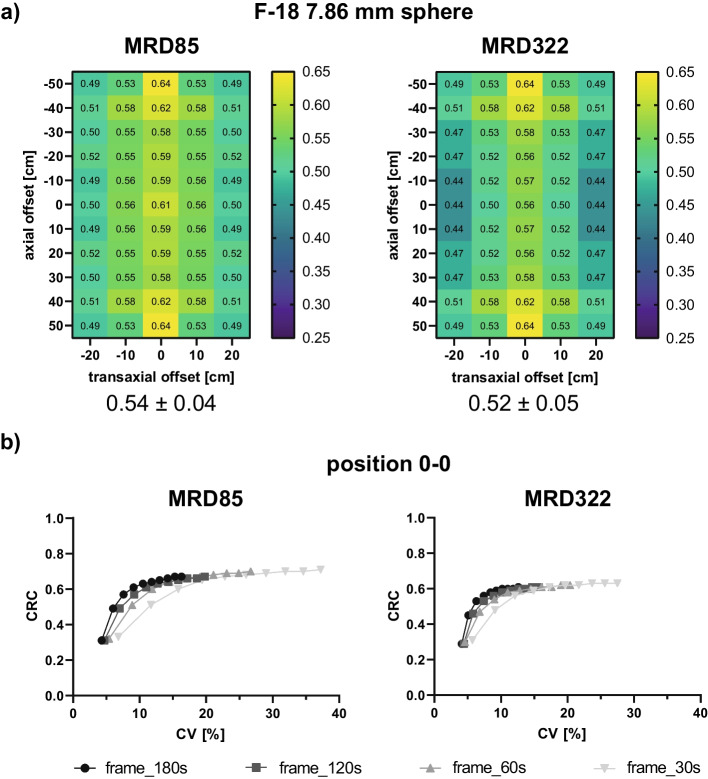
F-18 CRC maps along the FOV for the 7.86 mm sphere for MRDs of 85 and 322 (**a**), and CRCs as a function of CV at the cFOV position for different frame durations and MRDs (**b**). The mean ± standard deviation was determined over all positions (**a**). Each point represents one iteration, and the number of iterations increases from left to right (**b**)

Figure 4a presents the comparison of the 7.86 mm sphere CRC maps for MRDs 85 and 322, respectively. The CRC values decreased for MRD322 in the cFOV region with axial offsets up to 30 cm (e.g., cFOV: MRD85 = 0.61, MRD322 = 0.56). At axial offsets of 40 cm or more, CRC values were identical for both MRDs.

A distinct difference was detected in the noise characteristics when comparing both MRDs at the cFOV and for different frame durations (Fig. 4b). A lower CV was detected for MRD322 compared with MRD85 for all investigated frame durations. For the default clinical reconstruction setting with 4 iterations, CVs of 7.4% and 9.1% were determined for 180 s and MRD322 and MRD85, respectively. This difference increased with shorter frame durations (e.g., 30 s: MRD322 14.9%, MRD85 19.4%). Furthermore, the MRD85 reconstruction revealed higher CRC values than MRD322 (e.g., for 30 s and 4 iterations: 0.59 and 0.65 for MRD322 and MRD85, respectively).

**Fig. 5:**
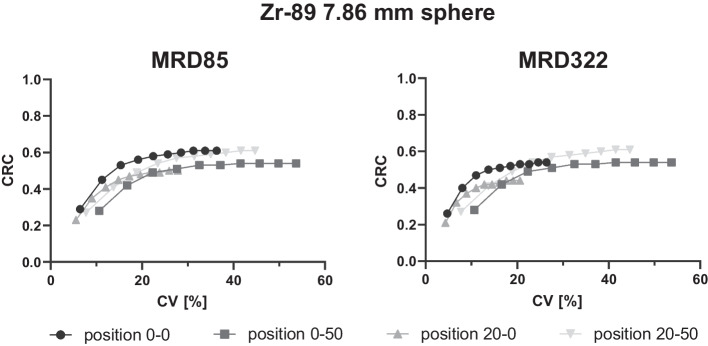
Zr-89 CRCs as a function of CV for MRDs of 85 and 322 for 4 different positions within the FOV. Position 0–0: axial and transaxial cFOV; position 0–50: axial offset of 50 cm, transaxial cFOV; position 20–0: axial cFOV, transaxial offset of 20 cm; position 20–50: axial offset of 50 cm, transaxial offset of 20 cm. Each point represents one iteration, and the number of iterations increases from left to right

For Zr-89, four selected positions were reconstructed with MRD322 (Fig. 5), as Zr-89 exhibited the largest voxel noise for MRD85. For the positions 0–0 and 20–0, a significant decrease in CV was detected compared with MRD85 for 4 iterations (position 0–0: MRD85 = 19.1%, MRD322 = 13.7%; position 20–0: MRD85 = 14.8%, MRD322 = 11.0%), whereas identical noise levels were detected for both positions at the axial edge (position 0–50: MRD85 & MRD322 = 27.6%; position 20–0: MRD85 & MRD322 = 23.4%).

## Discussion

This study investigated the PVE and voxel noise change throughout the 1.06 m axial FOV of the Biograph Vision Quadra PET/CT scanner. Phantom measurements were taken, and CRCs were determined at multiple positions within the FOV to characterize the PVE along the extended axial FOV for multiple isotopes. Furthermore, the voxel noise was assessed for four selected positions within the FOV with up to 10 iterations and the influence of the MRD on the PVE and noise was investigated.

The CRC values of the 37 and 28 mm spheres in the cFOV are comparable with reported data from the same scanner [7, 18], demonstrating similar system performance to other systems from the same vendor. Minor differences in CRC values may result due to VOI positioning, utilized software for analysis [19], as well as calculation of recovery (based on NEMA, etc.).

Furthermore, the measured CRCs of the 37 mm sphere in the cFOV are consistent with the results for the Biograph Vision PET/CT (Siemens Healthineers) reported by Reddin et al*.*, as both systems share the same technology [20]. Therefore, both systems have identical finite spatial resolution and image sampling, which determine the PVE [11, 21]; thus, contrast recovery in the cFOV is comparable.

The smallest sphere in the clinical NEMA standard International Electrotechnical Commission (IEC) body phantom has an inner diameter of 10 mm, slightly larger than the 7.86 mm sphere investigated in our study. Prenosil et al*.* reported a CRC of 74.4% for the 8:1 SBR and 64.3% for the 4:1 SBR for a 10 mm sphere size [7], which is higher than our results for the 7.86 mm sphere (8:1: 61.0%; 4:1: 46.8%). This difference can be attributed to the different sphere diameters and hence the larger PVE with respect to the spatial resolution of the system (3.35 mm full width at half maximum (FWHM) at 1/2 of the axial FOV and radial offset of 1 cm for MRD85 [7]).

As stated by Soret et al*.* [11], the PVE has a significant impact when the object size is smaller than 2 or 3 times the FWHM spatial resolution of the scanner, depending on the object shape, activity uniformity and other factors. Based on the determined spatial resolution of the Biograph Vision Quadra system [7], objects smaller than 9.57 mm would be affected by the PVE. This is consistent with our results demonstrating a significant decrease in CRCs for the 7.86 mm sphere compared with the larger sphere sizes and also explaining the difference relative to the CRC of the 10 mm sphere of Prenosil et al*.* [7]. Furthermore, the CRCs for the larger spheres exhibited a remarkable reproducibility with only minor changes along the FOV in contrast to the 7.86 mm sphere size. The PVE is more pronounced for voxel measurements at the edge of the spheres, as these are more affected by spill-in and spill-out into the background region. Therefore, the smaller sphere is more affected by the PVE than the larger spheres because the number of edge voxels is higher compared with the total volume of the sphere. Analyzing the 7.86 mm sphere data with a VOI with half of the actual sphere size centered on the sphere center and hence fewer edge voxels affected by the spill-in/spill-out to the background region revealed significantly increased CRCs (see Additional file 1: Fig S1).

The CRCs for the 7.86 mm sphere for F-18 increased at the axial edge (Fig. 2a, b and e) due to a reduction in the maximum oblique angle at this position, hence mitigating the degradation due to the parallax error, noncollinearity and Compton scatter.

The 7.86 mm sphere with Ga-68 and Zr-89 did not exhibit an increase in CRCs at the axial edge (Fig. 3e and f). Especially for Zr-89, a decrease in CRCs was detected when comparing the values at the cFOV and the axial edge (0.56 vs. 0.51), presumably because a larger noise was detected, affecting the accuracy of CRCs. Furthermore, the CRCs of larger spheres filled with F-18 did not increase at the axial edge, indicating that only smaller sphere sizes closer to the spatial resolution and detection limit exhibit this behavior.

Comparing our results to the uEXPLORER TB PET/CT scanner (United Imaging Healthcare, Shanghai, China) at UC Davis, the only other commercially available TB PET scanner approved for routine clinical use to date, revealed slight differences in CRCs. Spencer et al*.* reported a recovery of 95.8% in the cFOV for the 37 mm sphere compared with 88.3% in our work [6]. This difference is attributed to the smaller crystal size of 2.76 × 2.76 mm^2^ used in the uEXPLORER, resulting in an improved radial spatial resolution of 3.0 mm in the cFOV (radial distance 1 cm) [6] compared with the 3.2 × 3.2 mm^2^ crystal size of the Biograph Vision Quadra and a radial spatial resolution of 3.35 mm (at 1/2 of the axial FOV (53.0 cm) and radial offset of 1 cm [7]).

Comparing the 7.86 mm sphere CRCs with data of the same sphere size acquired on a preclinical dedicated Inveon PET system (Siemens Healthineers) revealed relatively comparable CRCs for the Quadra system. For the preclinical system, CRCs in the range of 54% to 82% depending on the reconstruction and correction algorithms used were detected for the 7.86 mm sphere with F-18 SBR 8:1 [22, 23], whereas the Quadra system revealed a recovery of 61% in the cFOV (Fig. 2a). Hence, although the spatial resolutions differ significantly between the two systems (Inveon: 1.63 mm FWHM at 5 mm radial offset, axial cFOV [24]; Quadra: 3.35 mm FWHM at 1 cm radial offset and at 1/2 of the axial FOV [7]), the comparable CRCs indicate the potential of the Biograph Vision Quadra as suitable for animal studies.

In general, comparisons of the CRC values along the axial FOV to the literature values are limited, as the NEMA protocol requires the phantom only to be centered in the FOV. Rausch et al. [18] recently investigated the CRCs for the Biograph Vision Quadra at four positions along the axial FOV (cFOV, axial offsets of 250 mm, 450 mm and 505 mm) and determined a stable behavior for the spheres larger than 13 mm and axial offsets up to 450 mm (for acquisition times of 120 and 600 s). This is consistent with our data reporting that for the 37 and 28 mm spheres the CRCs were stable along the axial FOV (see Fig. 2c and d).

Assessment of CRCs at one position is well suited for short axial FOV PET scanners (< 30 cm); however, with the increasing availability of TB PET/CT scanners, multiple phantom positions along the axial FOV should be considered to determine PVE changes.

Differences in CRCs of the 7.86 mm sphere for different isotopes (Fig. 2a, b, e and f) can be attributed to the different mean positron ranges in water (F-18: 0.6 mm, Zr-89: 1.3 mm, Ga-68: 3.5 mm [25]). Longer positron ranges result in a degradation of the spatial resolution [21, 26], which is consistent with our findings as the highest CRCs were determined for F-18 (Fig. 2a) and the lowest CRCs for Ga-68 (Fig. 2e). These results are also applicable to larger sphere sizes filled with Zr-89 and Ga-68, although the effect of the positron range might be slightly diminished due to the larger sphere sizes.

The determined changes in the PVE along the axial and transaxial FOV (Fig. 2) are nonnegligible and might substantially affect the quantitative analysis of patient data if no PVE correction is implemented. In particular, for TB PET scanners that enable simultaneous multiorgan investigations, this can be of high relevance. For example, for the gut-to-brain axis, both organs of interest will be placed at different axial (~ 60 to 70 cm apart) and transaxial positions. Depending on the positions inside the FOV, object size, the SBRs, count statistics and isotopes used, and according to the CRC maps (Fig. 2), this can result in an up to 50% difference between CRCs. This difference is especially crucial when comparing the uptake of different organ/lesion sizes and shapes, as well as target-to-background ratios, which are present when performing dynamic multiorgan investigations.

F-18 CV for the two larger sphere sizes were comparable to the 7.86 mm sphere with F-18 SBR 8:1 (e.g., cFOV, 4 iterations: 37 mm: 9.3%; 28 mm: 9.0%, 7.86 mm: 9.1%, Fig. 3). For the 7.86 mm sphere, CV was overall lowest for F-18 and Ga-68, whereas Zr-89 exhibited the largest CV of up to 54% (position 0–50, 10 iterations). The higher CV is caused by the lower count statistics of Zr-89 due to the low branching ratio of 22.7% [25]. Comparing the CV of the four investigated positions along the FOV revealed the largest CV for the position at the axial edge of the FOV (position 0–50) due to the decrease in sensitivity and count statistics at this position.

A CV < 15% is the acceptable tolerance level according to the ‘European Federation of Organisations for Medical Physics’ (EFOMPs) [27] for the NEMA IQ phantom. It must be noted, however, that the sizes of the phantoms investigated in our study are smaller compared with the NEMA phantom size to which the threshold applies, therefore potentially impacting the CV. Transferring this threshold to the phantom results from our study with 3 kBq/ml and 180 s acquisition time, the CVs for F-18 and Ga-68 (Fig. 3a-e) were below 15%, meeting the EFOMPs criterion for all investigated sphere sizes and SBRs for the default clinical reconstruction setting of four iterations. Furthermore, even for frame durations down to 30 s, the 7.86 mm sphere F-18 data (Fig. 4b) demonstrated CVs below 15%, meeting the EFOMPs criterion.

For Zr-89, the 15% threshold was exceeded at positions 0–0 (19.1%), 0–50 (27.6%) and 20–50 (23.4%). These noise characteristics are strongly dependent on the injected dose and especially for Zr-89 patient scans important to evaluate as a considerable lower dose is injected compared to [^18^F]-fluorodeoxyglucose ([^18^F]FDG) scans. To meet the threshold criterion, either larger filter kernels might need to be applied and/or an increase in the event statistics by a higher injected dose (though this might not be an option due to radiation dose) or longer acquisition time or reconstruction of the data with MRD322.

Reconstructing the data with MRD322 decreased the CV below 15% for position 0–0 (MRD85 19.1%; MRD322 13.7%) due to the increase in sensitivity. For the positions at the axial edge, the CV revealed no differences between the MRDs (MRD85 & 322: position 0–50 both 27.6%; position 20–50 both 23.4%). This is consistent with the sensitivity profile demonstrating no difference at the axial positions with a 50 cm offset to the cFOV between MRD85 and MRD322 [7]. However, especially for the positions closer to the cFOV, MRD322 can significantly enhance the SNR and CV for low-count studies such as late ImmunoPET imaging with Zr-89 [8], ultralow dose [28] and ultrashort imaging studies [29].

A direct comparison between MRD85 and MRD322 for the F-18 7.86 mm sphere (Fig. 4a) revealed lower CRC values for MRD322 in the cFOV region (axial offsets ≤ 30 cm and transaxial offsets ≤ 20 cm). This difference is presumably due to the degradation of the spatial resolution for MRD322 as more oblique LORs pronounce the parallax error. However, the increased count statistics for MRD322 led to less variation of the voxel values, which are used to determine the mean concentration and are thus considered to be more robust and reliable compared to MRD85. Similar CRCs were detected at the axial edge for both MRDs (offset 50 cm), which is consistent with the sensitivity profiles of both MRDs demonstrating similar sensitivities at the axial edge [7]. For the 37 mm sphere, only a minor difference was detected between both MRDs (Additional file 2: Fig S2), demonstrating the impact of noise on the 7.86 mm sphere CRCs.

## Conclusions

This work evaluated the PVE and voxel noise throughout the 1.06 m axial FOV of the Biograph Vision Quadra PET/CT. Distinct differences in the PVE within the FOV were detected for clinically relevant isotopes F-18, Ga-68 and Zr-89, as well as for different sphere sizes. Depending on the positions inside the FOV, the SBRs, count statistics and isotopes used, this can result in an up to 50% difference between CRCs. Hence, these changes in PVE can significantly affect the quantitative analysis of patient data. This effect is especially crucial when performing dynamic multiorgan investigations, in which the organs of interest are differently affected by the PVE based on their positions. Therefore, knowledge of the PVE change throughout the FOV should be included in the interpretation of quantitative results, especially in multiorgan studies, to enhance the scientific interpretation of these results.

Furthermore, reconstruction with full maximum ring difference (MRD322) resulted in slightly lower CRC values, particularly in the center FOV, whereas the CV significantly decreased compared with MRD85, which is especially important for low-count studies.

## Supplementary Information

**Additional file 1**.**Fig S1**: F-18 CRC maps along the FOV for the 7.86 mm sphere SBR 8:1. The VOI used for analysis was half the physical sphere size centered on the sphere center. Data were reconstructed with MRD85. The mean ± standard deviation was determined over all positions.

**Additional file 2**.**Fig S2**: F-18 CRC maps along the FOV for the 37 mm sphere for MRDs of 85 and 322. The mean ± standard deviation was determined over all positions.

## Data Availability

The datasets used and/or analyzed during the current study are available from the corresponding author on reasonable request.

## List of abbreviations

AC: Activity concentration
cFOV: Center field of view
CRC: Contrast recovery coefficient
CT: Computed tomography
CV: Coefficient of variation
EFOMP: European Federation of Organisations for Medical Physics
FWHM: Full width at half maximum
LOR: Line of response
LSO: Lutetium oxyorthosilicate
MRD: Maximum ring difference
NEMA: National Electrical Manufacturers Association
OP-OSEM: Ordinary Poisson ordered subsets expectation maximization
PET: Positron emission tomography
PSF: Point spread function
PVE: Partial volume effect
SBR: Sphere-to-background ratio
SiPM: Silicon photomultiplier
SNR: Signal-to-noise ratio
TB: Total-body
VOI: Volume of interest
