# Supplementary figures and images for: Characterization of the partial volume effect along the axial field-of-view of the Biograph Vision Quadra total-body PET/CT system for multiple isotopes
Source: EJNMMI Phys. 2023 May 27;10:33. doi: 10.1186/s40658-023-00554-7 (PMC10224893; doi:10.1186/s40658-023-00554-7)

# MRD85

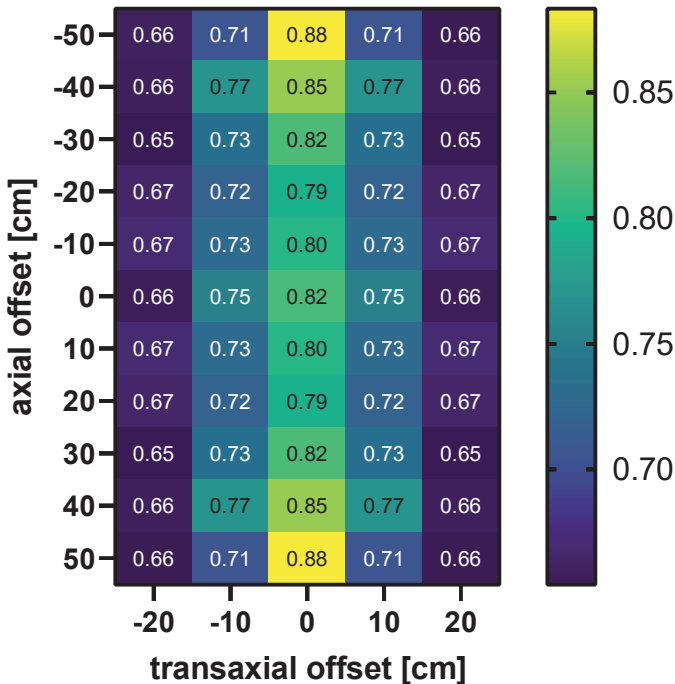

$0.72 \pm 0.07$

Supplement: Supplementary file 1 — Additional file 1.Fig S1: F-18 CRC maps along the FOV for the 7.86 mm sphere SBR 8:1. The VOI used for analysis was half the physical sphere size centered on the sphere center. Data were reconstructed with MRD85. The mean ± standard deviation was determined over all positions. [file 40658_2023_554_MOESM1_ESM.pdf]

# F-18 37 mm sphere

## MRD85

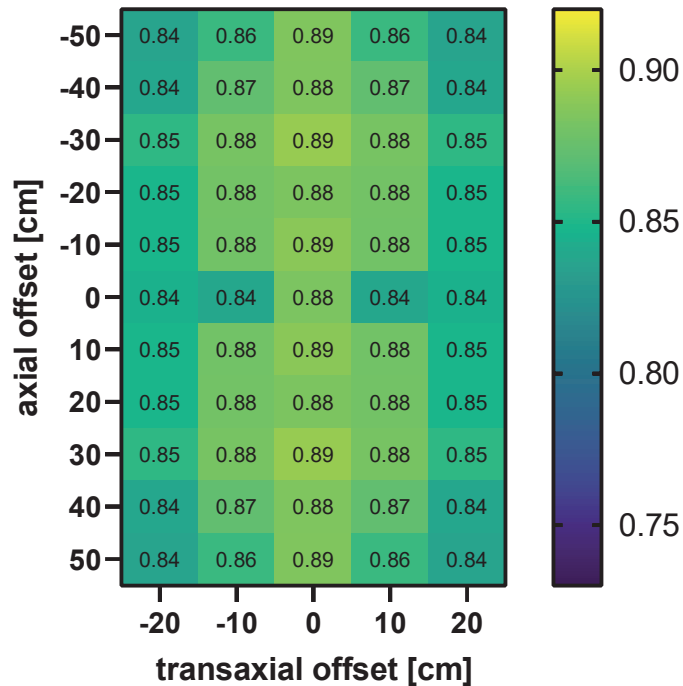

$0.86 \pm 0.02$

## MRD322

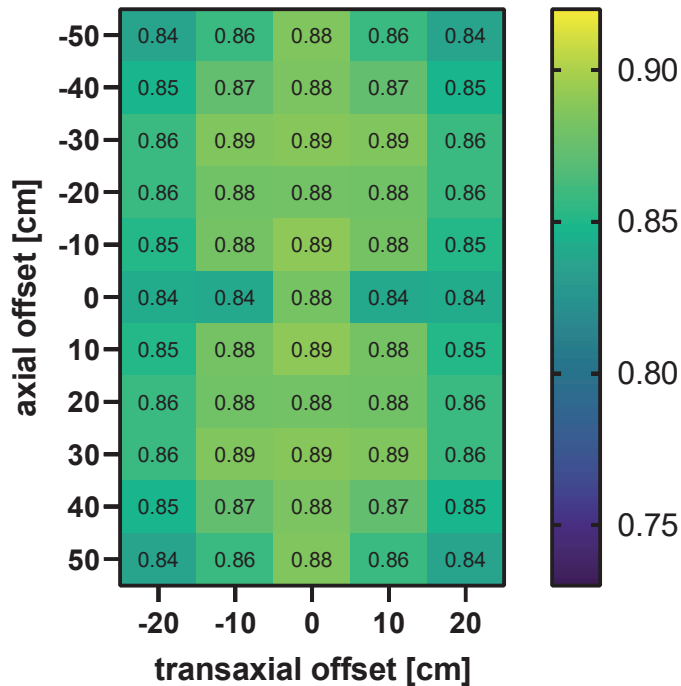

$0.87 \pm 0.02$

Supplement: Supplementary file 2 — Additional file 2.Fig S2: F-18 CRC maps along the FOV for the 37 mm sphere for MRDs of 85 and 322. The mean ± standard deviation was determined over all positions. [file 40658_2023_554_MOESM2_ESM.pdf]
